# Countering the poor oral health of people with intellectual and developmental disability: a scoping literature review

**DOI:** 10.1186/s12889-019-7863-1

**Published:** 2019-11-15

**Authors:** Nathan J. Wilson, Zhen Lin, Amy Villarosa, Peter Lewis, Philcy Philip, Bashir Sumar, Ajesh George

**Affiliations:** 1 School of Nursing and Midwifery, Centre for Oral Health Outcomes & Research Translation (COHORT), Western Sydney University, Hawkesbury Campus, Locked Bag 3, Richmond, NSW 2753 Australia; 2 School of Nursing and Midwifery, Western Sydney University, Hawkesbury Campus, Locked Bag 3, Richmond, NSW 2753 Australia; 3 COHORT, Western Sydney University, South Western Sydney Local Health District, Ingham Institute Applied Medical Research, Penrith, Australia; 4 Bangalore Baptist Hospital, Bellary Road, Hebbal, Bangalore, Karnataka State 560024 India; 5 School of Dentistry, Faculty of Medicine and Health, University of Sydney, Sydney, Australia; 6 Translational Health Research Institute, Campbelltown, NSW 2560 Australia

**Keywords:** Intellectual disability, Oral health, Health disparities, Dental interventions, Gender, Nursing

## Abstract

**Background:**

People with intellectual and developmental disability (IDD) have poor oral health and need support to maintain optimal oral health outcomes. Little is known about how, when and where to intervene for this population. Thus the aim of this review was to summarise the existing evidence surrounding improving oral health outcomes for people with IDD.

**Methods:**

A scoping literature review was conducted focusing on ‘oral health’ and ‘intellectual disability’. Systematic searches of five electronic databases were conducted in line with the study aims and two authors independently examined all records for relevance, with consensus achieved by a third author.

**Results:**

A small number of approaches and interventions were identified to support people with IDD to independently maintain optimal oral hygiene. Identified studies highlighted that caregivers play a vital role in the provision of oral health support, emphasising the effectiveness of educational interventions for caregivers. However, there was uncertainty regarding the efficacy of specific tooth brushing interventions for people with IDD. In cases of more severe IDD and/or dental-related behavioural problems, dental treatment under general anaesthesia was often both a necessary and effective method of oral health care provision. The findings also identified outreach and exclusive oral health services as successful strategies for increasing the limited access of people with IDD to oral care services.

**Conclusions:**

A uniform approach to supporting oral health for people with IDD is unlikely to succeed. A system-based approach is needed to address the diverse needs of the population of people with IDD, their caregivers and service context. Further high quality evidence is required to confirm these findings.

## Background

Recent evidence has highlighted the existence of health inequalities between people with intellectual and developmental disability (IDD) and people without IDD [1–3]. Of particular concern is the oral health of people with IDD, with research showing that this population has poorer oral health than those without IDD [4, 5]. In addition, a systematic review by Anders and Davis [4] has specifically shown that people with IDD have a higher prevalence of periodontal disease and untreated dental caries compared with people without IDD. The implications of poor oral health are substantial, with emerging research highlighting the impact poor oral health can have on general health, including significant associations with aspiration pneumonia and major chronic diseases such as cardiovascular disease, diabetes, respiratory disease and stroke [6–12]. Oral health also has an important influence on an individual’s psychological and social health. For example, poor oral health can lead to toothache, associated anxiety, difficulty performing daily activities, impaired social interactions and reduced nutritional intake [13–19].

People with IDD are particularly vulnerable to poor oral health and have more complex oral health care needs than people without IDD [20, 21]. This disparity is due to myriad risk factors that people with IDD may experience including barriers to accessing quality health care, the need for assistance with core activities such as oral hygiene, behavioural challenges, communication challenges, a higher prevalence of enteral feeding, and a higher likelihood of having lower educational and income levels when compared to people without IDD [22–24].

Caregivers, including paid care staff and family members, play a vital role in maintaining the oral health of people with IDD, particularly when they require assistance with core activities [4]. In recent years, following the shift from institutionalisation of people with IDD to community-based support, caregivers for people with IDD are often family members or support workers rather than trained health professionals [25]. Studies also revealed that lower caregiver education was associated with greater caregiver burden and less preventative dental care use [26]. Caregivers’ attitude towards oral health of special needs patients was found relatively unsatisfactory even though their knowledge was adequate [27]. If interventions to improve the oral health of people with IDD are to be implemented effectively, barriers faced by caregivers should be identified and carefully considered in the investigation and evaluation of these interventions. Likewise, barriers faced by mainstream oral health services also need to be an integral part of the solutions.

Some studies have identified a number of the barriers to oral health care as perceived by caregivers. Although a Cochrane review of oral health interventions published after our search was concluded reported low to very low certainty with all reported interventions [28], and another review explored indications for treatment under GA [29]^,^ there has been no broad-based review that also summarises and incorporates the range of barriers to effective oral health care as perceived by caregivers. Given the reported low to very low certainty in evidence, there remains a significant gap in our knowledge of when and how to intervene, given the heterogeneity of the population of people with IDD at different life stages.

The aim of this review was to compile current evidence regarding the promotion of better oral health in people with IDD in order to provide insight into the requirements of enhancing oral health outcomes for people with IDD. The objectives were to:

i) identify any published novel intervention studies and how they sought to provide solutions to poor oral health -for different people with IDD;
ii) explore the literature regarding the types of issues facing people with IDD, their caregivers and dental professionals
iii) identify non-clinical oral health care service contexts that are likely to be embedded in future practices

## Methods

### Design

A scoping review methodology was implemented to explore current studies, synthesise knowledge of the topic and identify gaps in the literature. This design was best suited to address the broad aims of the study and our desire to look beyond the small number of fully controlled interventions [28] in this area, and also because this design can incorporate a broad range of qualitative and quantitative studies [30].

### Search strategy

All articles relevant to the two study objectives were reviewed in this study using the following databases: Science Direct, PubMed, CINAHL, Scopus and Cochrane. Due to the differences in indexing between databases, each database had its own search strategy developed, including a combination of keywords used in conjunction with various Boolean operators, phrase searching, truncation and Medical Subject Headings. Key words used included: oral health, dental, caries, mental retardation, intellectual disability, learning disability, developmental disability, cognitive disability, intellectual impairment, mental deficiency, mentally defective, and psychosocial retardation.

### Selection process

**Fig. 1:**
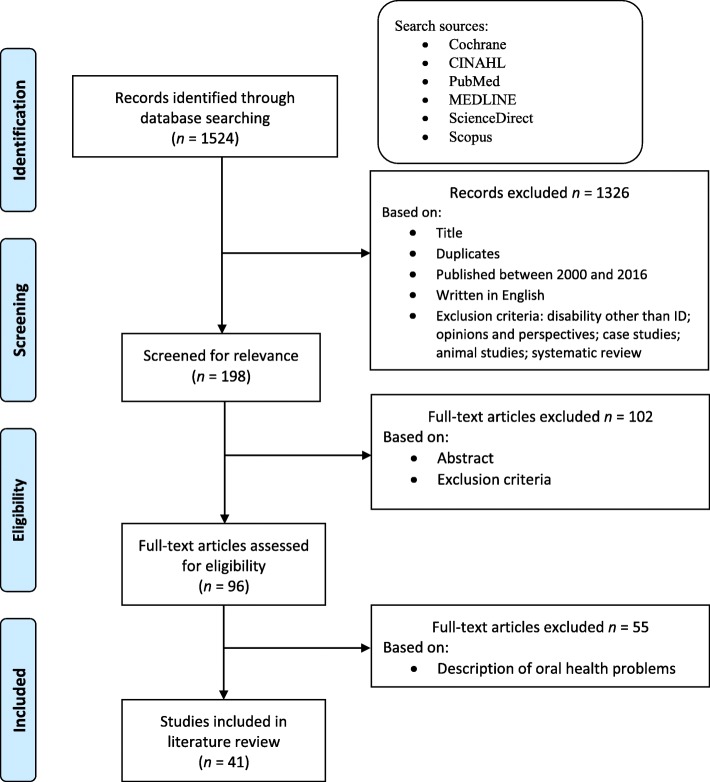
PRISMA for source selection

All records relating to the study aims published between January, 2000 and March, 2019, and written in English, were selected. Articles reporting on individuals with physical disability *only* were excluded from analysis. Descriptive studies including dental screening studies and oral health surveys that only reported on oral health status were also excluded. Articles were distributed among the team and independently reviewed for inclusion by pairs of investigators using a summary table, and in the case of conflicting opinions, a third reviewer was involved to achieve consensus. Although definitions varied, articles that reported on people with intellectual disability, learning disability, developmental disability, or IDD were all included provided the majority of participants were reported to have a primary diagnosis of IDD. An overview of this process using the PRISMA flowchart [31] is provided in Fig. 1.

## Results

In total, 41 papers were included in this scoping review; key information regarding authors, year of publication, study design, objectives/outcome measures, and key findings were extracted from each article and are summarised in Table 1. Most of the studies were from Europe (*n* = 12) followed by North America (*n* = 11), Asia/Arabia (*n* = 10), the UK (*n* = 3), Australia (*n* = 2), South America (*n* = 2), and Africa (*n* = 1). Using the Centre for Evidence-based Medicine’s research design flowchart [71], the majority of the studies used observational (*n* = 25) with the remainder using experimental designs. Studies are grouped into three categories to present a meaningful overview of the literature:

- Category 1: Interventions – technical and educational
  - 1a) tooth brushing
  - 1b) caregiver oral health education programs
  - 1c) dental care procedures, utilisation of general anaesthesia (GA), and sedation
  - 1d) dental clinic setting
- Category 2: Perspectives – people with IDD, caregivers, and contexts.
  - 2a) perspectives of people with IDD
  - 2b) perspectives of caregivers
  - 2c) perspectives of dentists
- Category 3: Non-clinical service delivery contexts

**Table 1:** Summary of reviewed oral health articles

Authors, Year (Country)	Design	Objective	Population	Key Findings
I. Interventions – technical and educational				
1a). Tooth brushing				
García-Carrillo et al., 2016 (Spain) [32]	Single-blinded cluster RCT	Compare effect of sonic powered versus manual toothbrush in adults with ID on plaque (PlI) and gingival (GI) indices.	64 adults with ID (male *n* = 34, female *n* = 30) in six clusters.	No significant difference in PlI or GI between techniques. No adverse effect or technical problems observed.
Shin & Saeed, 2013 (USA) [33]	Prospective cohort study	Explore most difficult tasks during tooth brushing and the effect of Oral Health (OH) education on technique.	14 children^a^ with ID from a disability-specific day program.	Most difficult tasks were brushing off identified plaque and brushing front teeth. Following education, greatest improvements were opening toothpaste and placing toothpaste on brush. No significant change in PlI.
Zhou, Wong & MacGrath, 2019 (Hong Kong) [34]	Two-phase pre-post intervention study	Examine the effectiveness of a visual-verbal integration model (VVIM) in training parents and children with IDD to dispense a pea-sized amount of toothpaste.	370 pairs of preschool IDD children (71.6% male) and their parents.	Children with higher adaptive skills were more likely to benefit from VVIM training. Parents should be motivated and reinforced to control toothpaste amount.
1b). Caregiver OH education programs				
Binkley et al., 2014 (USA) [35]	Cohort study	Measure effect of agreed carer action, training, environment changes and, ongoing coaching on paid caregivers’ self-efficacy, support provision, oral hygiene practice, and OH status of residents	21 paid caregivers (male *n* = 6; female *n* = 15) and 25 adults with ID (male *n* = 16; female *n* = 9).	No change in caregiver self-efficacy. Support provision to residents increased especially regarding supervision and environmental adaptation. Use of dental floss and disclosing solution increased in residents, but total time spent brushing teeth did not change. People with ID had a 50% plaque reduction.
Faulks & Hennequin, 2000 (France) [36]	Cohort study	Evaluate impact of OH education program on OH attitudes and behaviours.	Carers^a^ (*n* = 69 pre, *n* = 28 post) and residents^a^ (*n* = 67 pre, *n* = 25 post) from three centres for people with ID.	Frequency of teeth cleaning more than once a day rose by 28% (*p* < 0.05). More carers were able to clean all teeth of their residents (36% increase, *p* < 0.05), and 24% more found tooth cleaning easy (*p* > 0.05).
Fickert & Ross, 2012 (USA) [37]	Cohort study	Determine effectiveness of an OH educational program on paid caregivers’ knowledge and skills.	52 paid caregivers (male *n* = 7, female *n* = 45)	Oral hygiene knowledge significantly improved. Oral hygiene skills and compliance improved but this was not statistically assessed. Intervention may improve OH of people with ID requiring oral hygiene assistance.
Mac Giolla Phadraig, Guerin, & Nunn, 2013 (Ireland) [38]	Cluster RCT	Explore effect of OH train the trainer program on knowledge, behaviour, attitude and self-efficacy of paid caregivers.	25 care homes (*n* = 183) (21 trained (*n =* 159), 17 returned post questionnaire (*n* = 76))	Knowledge, reported behaviour and self-efficacy significantly increased among the intervention group. Multi-tiered training can be effective for caregivers.
Mac Giolla Phadraig, Guerin, & Nunn, 2015 (Ireland) [39]	Cluster RCT	Evaluate impact of OH train the trainer program on OH status and hygiene of people with ID in community based care homes.	76 caregivers (male *n* = 42, female *n* = 43) of adults with ID.	No statistically significant differences in PlI and the GI between control and intervention. Accuracy of outcome measures may be affected by difficulty of oral examination among people with ID.
Phlypo et al., 2018 (Belgium) [40]	Pre-post intervention study.	Explore effect of a dental student developed OH education program for caregivers in a residential facility for people with IDD.	Residents with severe to profound ID (intervention *n* = 18; control *n* = 19). Caregivers (intervention *n* = 12; control *n* = 22).	Change of PlI and GI from pre to post did not significantly differ between intervention and control. Intervention and control post GI scores were significantly different (*p* = 0.02).
1c). Dentist interventions - sedation, GA, and other procedures				
Chang, Patton, & Kim, 2014 (Korea) [41]	Cohort Study	Assess primary caregiver perceptions of the effect of dental treatment under GA on OH-related quality of life (OHRQoL) and family dynamics.	102 Primary family caregivers (male *n* = 66; female *n* = 36)	Percieved OHRQoL of patients with IDD was significantly improved by dental treatment under GA for patients older than 30 years, originally eating soft meals, displaying no or very low levels of cooperation, or receiving endodontic treatment.
Maeda et al., 2005 (Japan) [42]	Cross-sectional study	Explore if ICF codes can be used to assess tolerability of dental treatment for people with ID and determine its influence on application of GA or IVS.	Paid caregivers^a^ of 49 patients with ID (male *n* = 32; female *n* = 17)	The ICF codes were a useful guide for patient treatment tolerability and enabled easy interviewing.
Meurs, Rutten, & de Jongh, 2010 (The Netherlands) [43]	RCT	Assess effect of background information for patients with ID on level of cooperation during first dental visit.	57 patients with ID (male *n* = 40, female *n* = 17) (control *n* = 28, intervention *n* = 29)	No significant difference was found in patient cooperation between groups regardless of degree of ID and function.
Miyawaki et al., 2004 (Japan) [44]	Case-control study	Compare sedative doses required for patients with ID to those required for patients without ID.	73 dental patients with ID (male *n* = 63; female *n* = 10); 19 patients without ID (*n* = 19; male *n* = 8, female *n* = 11).	Required dose of propofol in subjects with ID was significantly higher than for other subjects. This could be attributed to the use of anticonvulsants in the ID group.
Mori, Amano, Akiyama, & Morisaki, 2000 (Japan) [45]	Descriptive	Evaluate effectiveness of a 6-week Professional Mechanical Tooth Cleaning Program (weekly cleaning using dental teeth cleaners and polishers) in improving OH and caries susceptibility.	Ten young adults with ID (male *n* = 9, female *n* = 1) with periodontal inflammation.	OH improved; probing depth and bleeding sites were reduced, however number of bleeding sites returned to baseline after 3 months. No change in susceptibility to dental caries. Mechanical tooth cleaning can be effective in improving OH for people with ID.
Sakaguchi et al., 2011 (Japan) [46]	RCT	Investigate use of propofol sedation using Bispectral Index (BIS)-guided target-controlled pump infusion (TCI) in dental patients with ID when compared to manual control	Intervention group: *n* = 20 (male *n* = 12; female *n* = 8) and Manual control group: *n* = 20 (male *n* = 15; female *n* = 5).	The BIS-TCI group had significantly reduced doses of propofol and shortened recovery times. BIS-TCI can reduce the dose and length of sedation for people with ID.
Silva et al., 2018 (Portugal) [47]	Observational study	Analyse the utility of Bispectral Index (BIS) for anaesthetic depth monitoring in paediatric dental patients with IDD.	17 patients with ID (female *n* = 7, male *n* = 10).	The BIS correlated with end-tidal sedative concentration and had good prediction probability except in patients with Lennox-Gastaut, West syndrome, cerebral palsy and epilepsy. BIS may be able to reflect anaesthetic depth in patients with some types of ID.
Vaessen, Schouten, van der Hoeve & Knape, 2017 (The Netherlands) [48]	Retrospective study	Evaluate safety and effectiveness of propofol sedation by trained nonmedical sedation practitioners in dental patients with ID in an office-based setting.	Participants^a^ with ID; mild (*n* = 21); moderate (*n* = 56) and severe (*n* = 47).	Propofol sedation was effective, allowing a sufficient sedation level without moderate or severe complications. The intervention allowed successful dental treatment in safe, familiar home surroundings.
Yeganegi & Tandon, 2008 (India) [49]	RCT	Assess the efficacy and retention of Fuji VII Glass Ionomer Cements (GIC) for surface protection in plaque-prone areas of teeth	100 patients with ID (male *n* = 48; female *n* = 52).	Mean plaque scores at follow up were significantly lower in the intervention group when compared to the comparison group. GIC helps to reduce plaque accumulation, although retention of the GIC on tooth surfaces was inadequate.
1d). Dental clinic setting				
Kim, Carrico, Ivey & Wunsch, 2019 (USA) [50]	Crossover RCT	Explore impact of a sensory adapted dental environment (SADE) on behavioural and physiological changes when compared to routine dental environment (RDE).	Children with developmental disability (male *n* = 14, female *n* = 8)	No significant difference in physiological scores (heart rate and oxygen saturation) between the two groups. The SADE group had significantly better observed behaviour scores compared to the routine dental environment group.
Potter, Wetzel & Learman, 2018 (USA) [51]	Cohort study	Examine whether a SADE had an effect on anxiety and agitation in adults with IDD when compared to RDE.	Adults with IDD (male *n* = 22; female *n* = 19) mainly with severe to profound IDD.	There was a significant reduction in the frequency and duration of observed agitation after SADE. There was also a significant reduction in anxiety after SADE as measured by heart rate and blood pressure.
Shapiro, Sgan-Cohen, Parush, & Melmed, 2009 (Israel) [52]	Crossover RCT	Examine the influence of a SADE compared to RDE on the behaviour and arousal levels of children with and without DD.	16 children with DD (male *n* = 11, female *n* = 5); 19 children without DD (male *n* = 13, female *n* = 6).	Both groups performed better in the SADE compared to the RDE, however the difference between the 2 environments was greater in children with DD.
Zhou et al., 2014 (UK) [53]	Descriptive study	Explore the applicability of the Verona coding definitions of emotional sequences (VR-CoDES) to assess emotional distress of patients with ID in a dental context.	14 patients with ID and complex communication needs (male *n* = 6, female *n* = 8).	Cues of emotional distress were reliably identified during seven of the 14 consultations. Although cues and responses were reliably identified, improving coding accuracy of responses is required through further guidance.
II. Perspectives – people with IDD, Caregivers, and Dentists				
2a). Perspectives of people with IDD				
Blaizot et al., 2017 (France) [54]	Qualitative study using focus groups (FG)	Explore ethical tensions in OH care management reported by adults with ID and then confirm these with family and paid caregivers	FG 1: Adults with ID^a^ (*n* = 8) FG 2: Family caregivers^a^ (*n* = 6) FG 3: Paid caregivers^a^ (*n* = 6)	Participants wanted a dentist competent to meet their specific needs, with a positive attitude toward people with ID, and the confidence to adjust their communication style for people with ID. Access barriers included the need for environmental adaptations, and cost.
Lees, Poole, Brennan & Irvine, 2017 (UK) [55]	Qualitative study	Explore experiences of people with ID and their carers who accessed community dental services.	Adults with ID^a^ (*n* = 4) an their caregivers^a^ (*n* = 6)	Participants valued the dental practitioners with ID-specific knowledge and positive attitudes. Dissatisfaction was attributed to poor communication, the transition form child to adult services and cost.
Mac Giolla Phadraig et al., 2016 (Ireland) [56]	Delphi study	Determine the priorities of people with ID regarding oral health services	Six participants (male *n* = 5, female *n* = 1) with mild-moderate ID.	Participants were disempowered in their interactions with dental services. They prioritised issues relating to control, empowerment and choice.
2b). Perspectives of caregivers				
Chadwick, Chapman & Davies, 2018 (UK) [57]	Descriptive phenomenological study	Identify factors influencing engagement of adults with ID in daily oral and dental care.	372 adults with ID (male *n* = 159, female *n* = 213)	Two global themes were identified: 1) Personal and lifestyle influences: physical, sensory, cognitive, behavioural and affective factors 2) Social and environmental factors: caregiver support, equipment/adaptations used and oral hygiene routine.
Eijsink, Schipper & Vermaire, 2018 (The Netherlands) [58]	Q-methodology	Explore all prevailing viewpoints that caregivers of institutionalised persons with ID have about oral hygiene.	Caregivers of people with ID living in institutions *n* = 40 (women *n* = 27).	Participants either: 1) recognised the consequences of poor dental care and took responsibility; 2) prioritised dental care as a way of promoting the social acceptability of their clients, 3) were highly motivated to promote OH but perceived obstacles; or 4) wanted to provide OH care but had insecurities about how to do so effectively.
Gerreth & Borysewicz-Lewicka, 2016 (Poland) [59]	Cross-sectional study	Evaluate unpaid caregiver views about access to and satisfaction with dental health care of their children with ID.	Parents/caregivers^a^ (*n* = 264) of children with ID.	31.8% parents/caregivers did not have any problems with access to dental care. The most commonly reported barrier to obtaining dental care was waiting time for a visit (36.7%). Most commonly, children were treated in dental surgery conditions (90.1%). Only 42.1% respondents were satisfied with their children’s dental care.
Kahabuka & Ndalahwa, 2006 (Tanzania) [60]	Cross-sectional study	Investigate oral hygiene practices and OH care given by parents of individuals with ID.	100 parents (male *n* = 48, female *n* = 52) of 100 individuals with ID (male *n* = 55, female *n* = 45)	65% of the individuals with ID were able to independently brush their teeth. Children who brushed less frequently experience significantly more bleeding. Few parents took their children to a dentist for dental pain (26%) or bleeding gums (37%).
Minihan et al., 2014 (USA) [61]	Cross-sectional study	Describe the experiences of caregivers (paid and family) supporting the at-home OH of adults with IDD.	Caregivers^a^ (paid *n* = 683; unpaid family *n* = 125) of adults with IDD.	Most adults with IDD required assistance with oral hygiene with behavioural problems interfering with OH care. Paid caregivers were significantly more confident in providing support.
Oliveira, Paiva, & Pordeus 2007 (Brazil) [62]	Cross-sectional study.	Analyse parental acceptance regarding physical and chemical restraint for patients with ID during dental care.	209 parents/legal guardians (male *n* = 44, female *n* = 165) of children with ID.	Participants were more likely to accept physical restraints for their child when parents were over the age of 35 and from an underprivileged economic class, and when the children had previously experienced physical restraints.
Pradhan, Slade, & Spencer, 2009 (Australia) [23]	Cross-sectional study	Compare access to dental care for adults with ID living in family homes, institutions and community housing.	484 caregivers (male *n* = 276, female *n* = 206) of adults with ID.	43.6% of respondents reported problems accessing dental care. Adults with ID living at home were more likely to have irregular dental visits than people with ID living in institutions.
Thole, Chalmers, Ettinger, & Warren, 2010 (USA) [63]	Cross-sectional study	Investigate the OH care activities and attitudes of care providers for people with ID.	138 paid caregivers (male *n* = 21, female *n* = 117).	Providing OHC to people with ID was rated as important to extremely important for 98% of respondents. Barriers include a lack of time and lack of staff. Behavioural issues were common (64.9%).
Versloot et al., 2008 (Canada) [64]	Cross-sectional study	Determine If the Dental Discomfort Questionnaire (DDQ) can identify dental pain in children with ID, and if two added items can increase its sensitivity.	Parents of 58 children with ID (male *n* = 40, female *n* = 18) completed the DDQ after dental examination.	There was a non-significant trend between having caries and parents reporting toothache. There was a significant association between mean DDQ scores and the presence or absence of caries. The standard DDQ has good predictive value in determining the presence or absence of caries and dental pain in children with ID.
Weckwerth et al., 2016 (Brazil) [65]	Cross-sectional study	To evaluate the parents’ perception of dental caries in children with ID.	Schoolchildren^a^ (*n* = 100) with (*n* = 50) without (*n* = 50) ID diagnoses, and their parents.	Both groups had a similar prevalence of caries free children. Parents of children with ID rated impact of caries on drinking, eating and pronunciation as more important than for parents of children without ID.
Wiener et al., 2016 (USA) [66]	Cross-sectional study	Explore if finances, employment and time burdens are associated with perceived need for and receipt of dental care.	Secondary analysis of 16,323 caregivers of children (male = 65.2%) who have CASD/DD/MHC.	Unmet need for preventative dental care was associated with employment and financial burdens of caregivers. Parents with either private or public health insurance policies were more likely to self-report that their children had all their OH needs met.
2c). Perspectives of dentists				
Byrappagari, Jung & Chen, 2018 (USA) [67]	Cross–sectional study	Examine level of access individuals with DD have to dental care and explore the dentists’ practices, attitudes and barriers to providing care to this population.	279 dentists (male 75.6%)	Most dentists provided care for people with DD (80.3%) and 58% were confident to do so. All dentists identified training and better reimbursement for services as key to improving care for these patients. Behaviour, inadequate training, and severity of patient’s condition were the most common reasons for not treating patients with DD.
Grant, Carlson, & Cullen-Erickson, 2004 (Australia) [68]	Phenomenological study	Explore the experiences and strategies involved in achieving positive OH outcomes for people with ID.	10 professionals^a^ (support worker *n* = 3; dental professional *n* = 4; other professional *n* = 3) caring for four adults with ID.	Direct support workers defined positive outcomes as acceptance of treatment, whereas professionals thought in terms of improvements in dental condition/oral hygiene. Strategies for better outcomes were consistent personnel and procedure, positive feedback to people with ID, exercising patience, and respecting their choices.
III. Non-clinical service delivery contexts				
Lo et al., 2004 (Hong Kong) [69]	Descriptive study	Describe the appropriateness, efficiency and acceptance of an OH outreach service for marginalised sub-populations including people with IDD.	1030 (male *n* = 618, female *n* = 412) adolescents and adults with ID	Mean numbers of decayed teeth were 1.8; mean numbers of missing teeth were 1.6. Problems with access, financial stress, and under-estimating the severity of the problem were the main barriers to OH service provision. The outreach service was viewed positively with more services and more frequent OH reviews requested.
Shyama, Al-Mutawa, Honkala, & Honkala, 2003 (Kuwait) [38]	Descriptive study	Evaluate the effectiveness of a school-based, supervised tooth-brushing program.	112 children with Down syndrome and ID (males *n* = 45, females *n* = 67).	Mean PlI and GI showed significantly decreased after the intervention. A supervised toothbrushing program can be effective in reducing PlI and GI.
York & Holtzman, 2004 (USA) [70]	Mixed methods design	Design and assess the effect of a school-based dental program for students with IDD.	Parents/guardians (*n* = 131) of students with IDD (male children *n* = 97, female children *n* = 34).	Two years after first dental visit, an increase was observed in parent/guardian consent, and the number of children seen by a dentist.

^a^participants’ gender not reported

*RCT* Randomised Controlled Trial

*ID* Intellectual disability

*PlI* Plaque Index

*GI* Gingival Index

*OH* Oral Health

*VVIM* Visual-Verbal Integration Model

*IDD* Intellectual and developmental disability

*DD* Developmental disability

*OHRQoL* Oral Health-Related Quality of Life

*ICF* International Classification of Functioning

*GA* General Anaesthesia

*IVS* Intravenous Sedation

*TCI* Target-Controlled pump Infusion

*BIS* Bispectral Index

*GIC* Glass Ionomer Cements

*COHIP* Child Oral Health Impact Profile

*FIS* Family Impact Scale

*SADE* Sensory Adapted Dental Environment

*RDE* Routine Dental Environment

*VR-CoDES* Verona Coding Definitions of Emotional Sequences

### Category 1: interventions – technical and educational

This category explored

#### 1a). Tooth brushing interventions (N = 3)

Shin and Saeed [33] explored which aspects of tooth brushing were more difficult for people with IDD than for people without IDD and whether instructions could improve their technique. They highlighted that the hardest tooth brushing steps were those requiring greater dexterity and comprehension, specifically brushing the front teeth (inside and outside) and removing plaque when identified from disclosing solution [33]. Although most of the instruction focussed on wrist rotation to better access brushing of all tooth surfaces, this did not significantly impact plaque index (PlI) and gingival index (GI). García-Carrillo et al. [32] compared the effect on PlI and GI using electronic versus manual toothbrushes, however found no significant differences in PlI and GI despite the sample with mild IDD reported to have appropriate fine motor skills [33]. Zhou et al. [34] examined if a visual-verbal integration model (VVIM) was effective at teaching children with IDD to dispense a pea-sized amount of fluoridated toothpaste. Their intervention showed that combining both visual and verbal prompts was effective in training parents and children with greater adaptive behaviour skills to dispense a pea-sized amount of toothpaste.

#### 1b). Caregiver oral health education programs (N = 6)

The study by Binkley et al. [35] used four strategies to capacity build paid caregivers, including: 1) agreed caregiver action using behavioural contracts; 2) capacity building through didactic (video and Powerpoint®) and observational (demonstration of teeth cleaning with a client) training; 3) environmental changes such as providing equipment and creating a calm atmosphere; and 4) ongoing coaching and reinforcement from a dental hygienist. In addition to a 50% decrease in the PlI, there was a positive change to caregivers’ oral hygiene supervision of people with IDD and their use of environmental adaptations. Faulks and Hennequin [36] involved both the client and the caregiver in their study. The program consisted of an oral presentation for paid caregivers about dental pathologies and the effect of plaque. This was followed by three repeated practical workshops where a dentist identified the presence of any plaque in each individual with IDD and then provided instructions on the best ways to support the oral health of that person with IDD. The results showed a significant increase in the amount of times teeth were brushed each day and in the caregivers’ ability to brush both the anterior and posterior teeth.

Fickert & Ross [37] used an existing education program where a dental hygienist spent four hours delivering a population-specific workshop, followed by a live demonstration and opportunities for the caregivers to practice their skills. At post-test and follow-up, there was a significant increase in caregiver knowledge and an improvement in oral health skills and compliance. The two Irish studies [38, 39] used a “train-the-trainer” model where managerial staff were trained by dental professionals; the managers then trained their staff. The training consisted of an oral presentation followed by practical workshops on plastic dummies and volunteers, and role-plays about complex oral health scenarios. At post-test, there was a significant increase in caregiver knowledge but no significant difference in caregiver self-efficacy between the intervention and control groups. The study by Phlypo et al. [40], reported on a dental student developed oral health program where the intervention group were provided with booklets, an information session and advice on appropriate toothbrushes and toothpaste. The only significant difference was in post-test GI between the intervention and the control group.

#### 1c). Dentist intervention: dental care procedures, utilisation of general anaesthesia (GA), and sedation (N = 9)

Two studies were based on dental procedures that were only available with access to a dentist (using glass cement on teeth and using a mechanical teeth cleaning/polishing tool) and, although reportedly beneficial to oral health, have few implications outside a dental setting [45, 49]. One study reported on dentists who were provided with background information about their patients with IDD to see if that improved the level of cooperation for more effective treatment, and whether they could better support the special needs of their clients throughout the dental consultation [43].

The other six studies provided insights into the factors that could affect dental routines and treatment procedures for people with IDD, including one that examined the appropriateness of implementing GA in dental treatment [41], four that focussed on the depth of sedation or GA administered [42, 44, 46, 47], as well as one retrospective study on the feasibility of propofol sedation for dental care [48].

Dental treatment under sedation or GA remains a treatment facilitator of choice for people with IDD and severe behavioural problems related to dental examination and treatment. A study conducted in Japan used the International Classification of Functioning (ICF) as a means to measure tolerability for dental procedures as a way of determining the suitability of a person for dental treatment under GA [42]. The study by Vaessen et al. [48] evaluated the safety and effectiveness of propofol sedation during dental treatment (*n* = 124); propofol sedation is effective providing its flexibility in addressing individual patient needs in combination with low-dose propofol [48]. Silva et al. [47] found that bispectral index (BIS) detected by a bilateral sensor placed on patient’s forehead may be able to reflect anaesthetic depth in patients undergoing dental treatment under GA. Despite the relatively small sample size, monitoring of BIS could potentially benefit some patients with IDD considering the difficulties faced by dentists in interpreting clinical signs of their neurological conditions under anaesthesia. Two of the Japanese studies sought to identify the ideal sedative dose for people with IDD; one study showed the sedative dose for people with IDD was consistently higher than in people without IDD [44], while the other study, using a measurement of the depth of sedation (BIS) in combination with a controlled infusion (TCI) of the sedative drug, reduced the overall amount of sedative drugs needed [46]. The study by Chang et al. [41] adapted the Child Oral Health Impact Profile (COHIP) and the Family Impact Scale (FIS) into short forms COHIP-14 and FIS-12 to examine the impact of dental treatment under GA, with a preoperative questionnaire administered on the day of scheduled treatment and a postoperative questionnaire completed within 1 week to 1 months’ time by caregivers of patients with IDD. Their findings highlighted that dental treatment under GA was worthwhile given the improvement of carer-perceived Oral Health-related Quality of Life (OHRQoL), despite reported restrictions on time and the known additional costs of procedures under a GA.

#### 1d). Dental clinic setting (N = 4)

A sensory adapted dental environment (SADE) is where the provision of sensory modifications within the clinical context is modified to reduce dental anxiety for people with IDD. The crossover Randomised Controlled Trial (RCT) by Shapiro et al. [50], modified visual, tactile, somatosensory and auditory stimuli in a dental clinic for 16 children with IDD and 19 typically developing children. They revealed that children with IDD showed higher levels of relaxation and cooperation in the SADE compared with routine dental environment (RDE). Similarly, two other recent studies observed the efficacy of SADE on dental anxiety of children with IDD (*n* = 22) [50] and adults with IDD (*n* = 41) [51]. Potter et al. [51] added to the evidence with both reduced frequency and duration of agitated behaviours in the SADE condition, whereas Shapiro et al. [52] only recorded the duration. The study by Kim et al. [50] involved pre-appointment sensory/behaviour assessment and individualised sensory modifications, which also received positive feedback from the participants. It is also important for health care workers to be able to identify the verbal and non-verbal cues of emotional distress of these patients promptly. Zhou et al. [53] used the *Verona coding definitions of emotional sequences* (VR-CoDES) to assess distress in the dental context, through reviewing 14 dental consultation videos from patients with varying degree of IDD. Their findings indicate that VR-CoDES is a potentially reliable tool for understanding and managing emotional distress of dental patients with complex communication needs.

### Category 2: perspectives – people with IDD, caregivers, and context

#### 2a). The perspectives of people with IDD (N = 3)

Mac Giolla Phadraig et al. [56] explored, using a Delphi process, what people with IDD viewed as being the most important components of a dental visit. Having an informed dental workforce with insight into the issues facing people with IDD was ranked as the most important and cost was the lowest ranked issue. This group of people with IDD were potentially disempowered in their interactions with dental services about their dental care as they either did not, or perhaps could not, complain if unhappy with the services and who were unlikely to have the means or capacity to choose alternative services. Suggestions to improve access to and the quality of services included both services and people with IDD being involved in shared decision-making and having access to more meaningful information.

Lees et al. [55] conducted individual semi-structured interviews with adults with IDD (*n* = 4) and their carers (*n* = 6) to explore their community dental services experiences. Although the level of satisfaction across multiple dental service domains was high, the communication problems in the movement from child to adult services were noted. The focus group study by Blaizot et al. [54] first analysed interviews with people with IDD (*n* = 8) to generate themes and subsequently used the emergent themes to organise information from the other two focus groups: family caregivers (*n* = 6) and paid caregivers (*n* = 6). Both studies highlighted the discrepancies in communication competencies of dental professionals, as well as barriers to accessing affordable dental services.

#### 2b). Caregiver perspectives and suggestions to improve oral health (N = 11).

Barriers reported by caregivers included factors associated with the person with IDD, such as lack of comprehension regarding why oral care is essential [57], noncompliance with oral hygiene care [61–63] and to alleviate anxiety around oral care [57, 64]. Suggestions for improving oral health outcomes included the provision of specialised dental clinics in disability services [59], access to specific support for culturally negative attitudes towards IDD [60] and training for unpaid caregivers, using tools such as the Dental Discomfort Questionnaire (DDQ) to help identify dental pain earlier [60], and individualised training that targets specific behavioural challenges [63].

Other suggestions were associated with social and environmental support for caregivers. Four studies reported that caregivers recognised the importance of delivery of oral care, but also their self-reported incompetence and lack of training [57, 58, 60, 61]. In the USA, paid caregivers were reported to be more confident in providing oral health care and support when compared to unpaid caregivers [61] and in Australia, people with IDD were more likely to have regular dental visits if they lived in residential services compared to people with IDD living at home [23]. Lack of access to skilled dentists working with adults with IDD was another significant factor [23]. Finances, employment and time burdens were also reported to hinder the oral care practices from these caregivers [63, 66]. The national survey by Wiener et al. [66] analysed responses from caregivers of children with Autism Spectrum Disorder, Developmental Disability and Mental Health Conditions (CASD/DD/MHD) (*n* = 16,323) in the USA, which reported the association between unmet needs of preventive dental care and financial burdens of the caregivers.

#### 2c). Perspectives of dentists or other health professionals (N = 2).

Byrappagari et al. [67] examined the perspectives of dentists (*n* = 291) in Michigan, USA of providing care to people with IDD. The results indicated the following barriers: 1) behaviour management problems; 2) inadequate training/experience; 3) severity of patient’s condition; 4) inadequately trained staff; and 5) the additional time required to treat versus inadequate reimbursement. This study reflected the support needed for general dentists in providing better dental care for people with IDD and could potentially contribute to continuing education for practising dentists. Grant et al. [68] reviewed a number of professional viewpoints on how to best achieve better oral health outcomes for people with IDD. Noted strategies were consistent personnel over time, positive verbal feedback to the person with IDD, and adapting a more patient communication style.

### Category 3: non-clinical service delivery context (*N* = 3)

Two studies reported on school-based programs [72, 70] and one on a community outreach program [69]. The school-based dental programs not only reduced many of the access barriers to seeing a dentist, but also improved PlI and GI in children with IDD [72, 70]. Access barriers were also addressed in the outreach program in Hong Kong where repeat visits in the future were requested [69].

## Discussion

This study is the first to examine in detail reported strategies to counteract the poor oral health of people with IDD. It has highlighted several aspects of oral health care for people with IDD. One is that the efficacy of specific toothbrushing interventions for people with IDD is yet to be established. Another is that the role of caregivers in the provision of oral health support is vital. Further, for people with more severe IDD and/or dental-related behavioural problems, dental treatment under GA is often both a necessary and effective means of providing oral health care and of improving oral health. Access to services and educational supports remains difficult for people with IDD and their caregivers, however outreach and exclusive services appear to be successful strategies for increasing access. Finally, a range of educational interventions for caregivers are reportedly effective. However, unpaid caregivers do not always have immediate access to these. The remaining gaps in our knowledge are: 1) ideally, where and when should health professionals intervene in oral health care for people with IDD, 2) how should intervention differ when supporting different sub-populations of people with IDD, different caregiver groups, and different service contexts, and 3) how should people with IDD who present to specialised services with severe intolerance to oral health support be best cared for?

### Insights into interventions

This review highlighted two types of educational interventions; those that were designed for people with IDD and those designed for paid caregivers. That there were no significant differences in oral health outcomes between the manual and electronic toothbrushes in the study by García-Carrillo et al. [32] suggesting, at least for this sample of people with mild IDD, that technique was not the issue. What was unclear in the literature reviewed was an identification of specific variations between a procedural intervention (e.g., how to approach the task of teeth cleaning conceptually and how to remember to clean one’s teeth) and a technical intervention (e.g., how to technically execute the task of holding a toothbrush and cleaning one’s teeth). That is, the interventions reported were about technique and did not include any strategy for promoting independence, for example, by providing daily reminders or visual toothbrushing charts as suggested for children [73].

It is likely that most toothbrushing interventions would be best targeted at people with the capacity to understand and follow instructions without any associated physical disability that preclude task performance. There would also be the sub-population of people with IDD who had the potential to be semi-independent and would likely require a combination of oral health supports such as toothbrushing interventions and the employment of caregivers trained in the provision of oral health support. Although contributory factors to independence are not clearly reported in the literature, the paper by Maeda et al. [46] about using the ICF as a functional framework to identify differing levels of tolerance to dental procedures is one potential starting point for identifying how to target interventions to individuals.

The interventions reported for paid caregivers all described significant outcomes and were broadly based on two principles; those of enhancing theoretical knowledge and providing opportunitities for caregivers to practice alongside an expert, and offering regular reinforcement over time [35–39]. Two things remain unclear however: i) the length of time the effects of the educational interventions last; and ii) how unpaid family caregivers could access these educational opportunities. The first issue is vital; just because caregivers know how to clean teeth, does not mean that they know the value of oral hygiene [27]. Staff motivation to provide support that is enabling and of high quality differs depending on organisational culture [74, 75]. Disability services require their staff to undertake mandatory training in a range of different areas. The opportunity might exist for oral health educational interventions to be embedded into annual mandatory staff training to promote the value of and potential for better oral health outcomes.

Answers that might identify how to best help unpaid family caregivers access and pay for oral health training are not clear from this review; different funding and service access structures across the world means that overcoming these barriers would be country-specific. In the Australian context, the new National Disability Insurance Scheme (NDIS) has shifted funding to the individual and although it has raised a number of issues around workforce development [76]. For the first time this change offers a concrete way for family caregivers, theoretically, to access specific training in matters of health care such as oral health. As far as we are aware, however, no such training actually exists in Austalia. Nevertheless, the provision of funding codes related to health procedure training for caregivers holds promise, and should be explored further [77]

### Insights into service contexts

Some dental interventions for people with IDD can only be provided in the context of a specialised disability dental clinic with GA facilities [41] and where behavioural support options have been exhausted. However, these are a specialised and expensive service option that realistically can only be provided in larger hospital/clinic settings where appropriate perioperative supports are available.

One unintended consequence of the provision of exclusive and specialised dental services could be that limited resources are allocated to providing extreme supports leaving gaps in other mainstream services. Mainstream oral health services should ideally be positioned to make whatever reasonable adjustments are necessary to promote access and inclusion of people with IDD [78]. The use of sensory adpated environments and increasing the knowledge base of dentists and their ancillary staff about IDD, are both important capacity-building strategies that should be implemented more widely [52]. Indeed, people with IDD want to access dental services which have an appreciation of their unique needs [56]. In addition, specialist dental outreach services [69] provide one example of a systems-based approach where adapting centre-based clinical models of dental care in partnership with community-based services, improves access and improves outcomes. This outreach model also mirrors the decentralised “hub and spoke” model for community therapy services (OT, speech, physiotherapy) for people with IDD in rural Australia where the main therapy teams are based in a major centre, but provide outreach services in smaller towns [79].

### Implications for research and practice

The authors have not clearly identified at which point intervention is likely to be most beneficial and cost-effective for the promotion of oral health amongst people with IDD. A combination of effectve interventions might have to be developed for implementation at the differing degrees of function, caregiver type, and with dental health professionals to maximise the likelihood that oral health can be maintained and improved. The promising educational interventions suggest that an opportunity exists for researchers, clinicians, and caregivers to collaborate on a standardised, yet adaptable, education program for caregivers in various contexts of care delivery. An example of the types of interventions we are referring to include nurse-led oral health support and training programs for the caregivers of people with IDD who also have chronic and complex health problems that directly impact upon the provision of daily oral hygiene. For instance, oral health support to a person with IDD who has a tracheostomy or is at risk of aspiration. Another could be led by a dental officer and targeted at people with milder degrees of IDD and the potential to be independent with their daily oral hygiene. A third example could be led by a health worker who provides home-based early intervention training for the families of people with IDD who present with behavioural challenges impacting upon their oral health. Although specific to the Australian context, such interventions would fit within the current schedule of services as defined by the NDIS [79].

What is less clear from our review, however, is how the causes of poor oral hygiene, and subsequent interventions to resolve the same, vary within and between contexts. In particular, the sheer range of different systems and eligibility criteria for dental services across the world makes it almost impossible to generalise. Nevertheless, a systems-based approach to oral hygiene for people with IDD that incorporates procedural, behavioural and educational elements and that is adaptable enough to be applied in a variety of client care contexts needs to be developed through an ongoing program of rigorous research.

### Limitations

This is a small and diverse body of work and while the review has highlighted a small number of potential areas to focus future interventions, more questions arise. Nevertheless, it presents a starting point for an applied research agenda in this area using the published peer reviewed literature from the selected databases. Another limitation is the use by some authors of vague disability identifiers, such as ‘special needs’ and ‘learning difficulty’ that were excluded as it was not certain the study was about people with IDD. Further, the inability to compare participant groups across studies due to different definitions, demographic profiles and support needs is limiting. The lack of a critical appraisal of included studies represents another limitation, however, the lack of controlled intervention studies in this area, the diverse range of methods, and different types of papers published meant that such a review would offer little to our conclusions. The exclusion of grey literature is another limitation, however adding government and professional reports of guidelines from around the globe is not feasible in a review of this nature.

## Conclusion

People with IDD have poor oral health and there is no clear systems-based approach or intervention framework that takes full account of the diverse support needs of the population. What is clear is that a one-size-fits-all approach is unlikely to be effective at maintaining or improving the oral health of people with IDD who are least able to participate in their own care. While educational and technical interventions can be developed and implemented with support from caregivers to people with milder degress of IDD, the problem of caring for the oral health of people with more severe IDD, who are dependent, and who have an aversion to oral health care interventions as fundamental as regular teeth cleaning, remains unresolved. People with IDD, service providers, and paid and unpaid carers need to balance the distribution of educational and technical resources amongst people with IDD who are most able, those who are least able, and all those who fit in between.

## Data Availability

All data generated or analysed during this study are included in this published article (and its supplementary information files).

## Abbreviations

BIS: Bispectral Index
CASD/DD/MHD: Children with Autism Spectrum Disorder, Developmental Disability and Mental Health Conditions
COHIP: Child Oral Health Impact Profile
DDQ: Dental Discomfort Questionnaire
FIS: Family Impact Scale
GA: General Anaesthesia
GI: Gingival Index
ICF: International Classification of Functioning
IDD: Intellectual and Developmental Disability
OHRQoL: Oral Health-Related Quality of Life
PlI: Plaque Index
RDE: Routine Dental Environment
SADE: Sensory Adapted Dental Environment
VR-CoDES: Verona Coding Definitions of Emotional Sequences
VVIM: Visual-Verbal Integration Model
